# Histone lysine methylation modifiers controlled by protein stability

**DOI:** 10.1038/s12276-024-01329-5

**Published:** 2024-10-11

**Authors:** Sungryul Park, Jin Hwa Cho, Jeong-Hoon Kim, Jung-Ae Kim

**Affiliations:** 1 Disease Target Structure Research Center, Korea Research Institute of Bioscience and Biotechnology, Daejeon, South Korea; 2 Department of Bioscience, University of Science and Technology, Daejeon, South Korea; 3 Aging Convergence Research Center, Korea Research Institute of Bioscience and Biotechnology, Daejeon, South Korea

**Keywords:** Ubiquitylation, Ubiquitylation, Histone post-translational modifications

## Abstract

Histone lysine methylation is pivotal in shaping the epigenetic landscape and is linked to cell physiology. Coordination of the activities of multiple histone lysine methylation modifiers, namely, methyltransferases and demethylases, modulates chromatin structure and dynamically alters the epigenetic landscape, orchestrating almost all DNA-templated processes, such as transcription, DNA replication, and DNA repair. The stability of modifier proteins, which is regulated by protein degradation, is crucial for their activity. Here, we review the current knowledge of modifier-protein degradation via specific pathways and its subsequent impact on cell physiology through epigenetic changes. By summarizing the functional links between the aberrant stability of modifier proteins and human diseases and highlighting efforts to target protein stability for therapeutic purposes, we aim to promote interest in defining novel pathways that regulate the degradation of modifiers and ultimately increase the potential for the development of novel therapeutic strategies.

## Protein stability in epigenetics: targeting modifier degradation pathways

Histone modifications, such as methylation, are key in controlling gene expression by changing the structure of chromatin, the DNA and protein mix in our cells’ nucleus. This study investigates how the stability of histone lysine methylation modifiers—enzymes that add or remove methyl groups from histones—is managed. It’s a study aimed at understanding the delicate balance of these modifiers in the cell. The researchers studied various cell processes, including the ubiquitin-proteasome system, and post-translational modifications, that affect the stability of these enzymes. The results show changing the stability of these modifiers can alter histone methylation patterns, suggesting new ways to target diseases like cancer. Researchers conclude that understanding the control of enzyme stability offers a promising path for developing therapies that can correct abnormal gene expression by targeting the enzymes responsible for histone modifications.

This summary was initially drafted using artificial intelligence, then revised and fact-checked by the author.

## Introduction

Lysine methylation in core histones—H2A, H2B, H3, and H4—comes in three ‘flavors’: monomethylation (me1), dimethylation (me2), and trimethylation (me3). Different genomic regions harbor distinctive types of histone lysine methylation modifications, each with specific functional implications. Trimethylation of histone H3 at lysine 27 (H3K27me3) and di- or trimethylation of H3 at lysine 9 (H3K9me2/3) function to suppress transcription from facultative and constitutive heterochromatin, respectively. Trimethylation of H3 at lysine 4 (H3K4me3) facilitates transcriptional initiation and elongation, whereas trimethylation of H3K36 (H3K36me3) and H3K79 (H3K79me3) are involved in efficient transcriptional elongation. In addition, histone lysine methylation affects genomic integrity by regulating DNA repair activity and the cell cycle^1^.

**Fig. 1:**
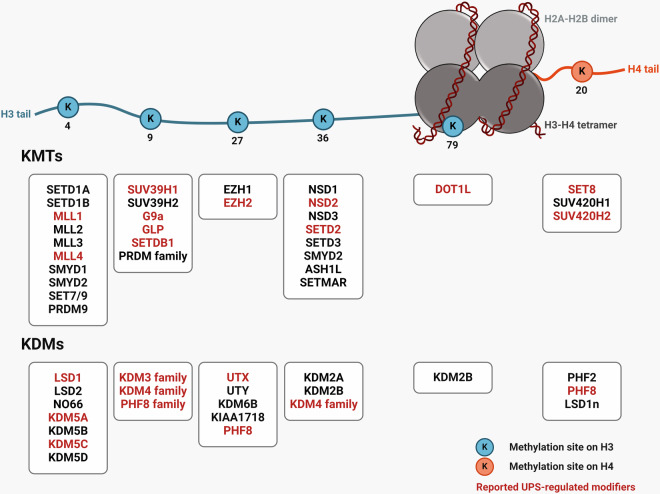
Histone lysine methylation modifiers. Schematic representation of a nucleosome showing the principal lysine methylation sites on histones H3 and H4. The histone octamer composed of an H3-H4 tetramer combined with two H2A-H2B dimers, along with the DNA lesion surrounding them, collectively constitute the nucleosome, which is the main structural element of chromatin. The reported KMTs and KDMs for each type of lysine methylation are indicated. Modifiers whose protein stability is reported to be regulated by the UPS are highlighted in red.

The methylation status of histones at different lysine residues is controlled by the reciprocal actions of specific histone lysine modifiers: histone lysine methyltransferases (KMTs) and histone lysine demethylases (KDMs) (Fig. 1). The different activities of KMTs and KDMs in a given cellular context play crucial roles in modulating chromatin architecture through histone lysine methylation. The discovery that many nonhistone methylation modifications are also catalyzed by KMTs and KDMs further underscores the importance of these modifiers beyond their roles in chromatin dynamics^2^. Dysregulation of the activity of specific KMTs or KDMs is often linked to diverse pathological conditions, including cancer and neurodegeneration^3,4^. In recent decades, targeting malfunctioning KMTs and KDMs has emerged as a powerful therapeutic strategy. Extensive efforts have led to the development of several inhibitors of histone lysine methylation modifiers, most of which directly interfere with the catalytic activity of the targeted modifier^5^. However, the conserved amino acid composition and structural similarities in the catalytic domains of modifiers, such as the su(var)3-9, enhancer-of-zeste and trithorax (SET) domains in members of the KMT family and the jumonji C (JmjC) domains in members of the KDM family, make the development of specific catalytic inhibitors challenging.

Ubiquitin‒proteasome system (UPS)-dependent protein degradation performs crucial functions in diverse cellular activities^6^. Polyubiquitination—a signal for protein degradation—occurs in sequential steps dependent on ubiquitin-activating enzymes (E1), ubiquitin-conjugating enzymes (E2) and ubiquitin ligases (E3). Mammalian genome encodes more than 600 E3 ligases. On the basis of the structural features involved in ubiquitin transfer mechanisms, E3 ligases are categorized into three classes: really interesting new gene (RING), homologous to E6AP C-terminus (HECT) and RING-between-RING (RBR) ligases. A subset of RING E3 ligase members form complexes, such as cullin-RING ligases (CRLs), that ubiquitinate substrate proteins^7^. Another multisubunit E3 ligase complex is anaphase-promoting complex/cyclosome (APC/C), which is a complicated assembly of 19 subunits, including a RING subunit (APC11) and a cullin-like subunit (APC2). Conversely, deubiquitinases (DUBs) remove ubiquitin moieties from ubiquitin-conjugated proteins or polyubiquitin chains, thereby increasing the stability of the target protein^8,9^. The human genome encodes ~100 DUBs, which are broadly classified as cysteine proteases or metalloproteases^10,11^.

**Fig. 2:**
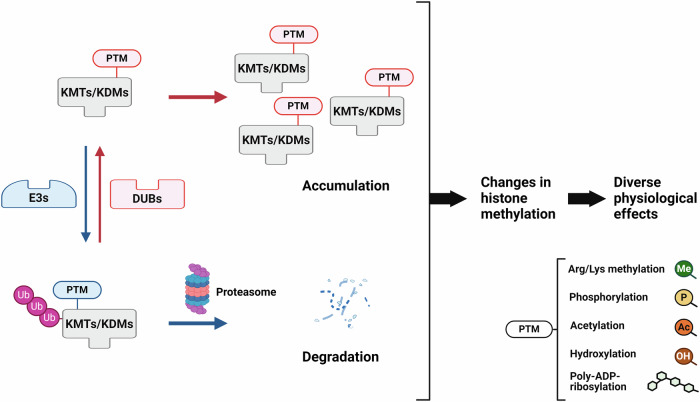
The UPS-mediated regulation of KMTs/KDMs. Schematic representation of the UPS-mediated regulation of histone lysine methylation modifiers. Polyubiquitin chains are conjugated to KMTs/KDMs via E3 ubiquitin ligases. The resulting polyubiquitinated proteins are subsequently recognized by the proteasome and degraded into small peptides and amino acids. Conversely, polyubiquitin chains can be disassembled by DUBs, stabilizing the KMTs/KDMs. Various PTMs, including methylation, phosphorylation, acetylation, hydroxylation and poly(ADP-ribosyl)ation, affect the ubiquitination and deubiquitination of KMTs/KDMs. The dynamic regulation of KMT and KDM protein stability changes the status of histone methylation and thereby mediates diverse physiological effects.

The stability of histone lysine methylation modifier proteins is under the control of distinct regulatory mechanisms. Interactions with particular ubiquitin ligases and/or DUBs are known to mediate the proteasomal degradation of these modifiers. In addition, diverse posttranslational modifications (PTMs) of modifiers control the stability of these proteins via distinct mechanisms (Fig. 2). Accordingly, harnessing protein degradation pathways that regulate individual histone lysine methylation modifiers could be a promising approach for developing inhibitors specific for individual modifiers.

Accumulating evidence has revealed various mechanisms by which the stability of specific histone lysine methylation modifier proteins is regulated. Here, we review the current understanding of how the degradation of individual histone lysine methylation modifiers is regulated by particular UPS pathways. We also discuss diverse molecular and cellular signaling pathways that regulate the stability of modifiers in a UPS-dependent manner, including the installation of different PTMs. Additionally, we highlight the clinical importance of regulating the stability of modifier proteins in controlling histone lysine methylation dynamics, summarizing recent efforts to develop strategies for the targeted degradation of these proteins, with the ultimate goal of manipulating the histone lysine methylation status in the context of human diseases.

## Protein stability of H3K4 methylation modifiers

The members of the Set1 family, which includes SET domain containing 1A (SETD1A)/KMT2F, SETD1B/KMT2G, mixed-lineage leukemia 1 (MLL1)/KMT2A, MLL2/KMT2B, MLL3/KMT2C and MLL4/KMT2D in mammals, function as H3K4 methyltransferases. Each Set1 family member forms a large complex with common interacting subunit proteins known as WRADs, which are composed of WD repeat domain 5 (WDR5); retinoblastoma-binding protein 5 (RBBP5); absent, small or homeotic-2 like (ASH2L); and Dpy-30 histone methyltransferase complex regulatory subunit (DPY30)^12^. In the presence of WRADs, MLL1 and MLL2 catalyze H3K4me1/2/3, whereas MLL3 and MLL4 catalyze H3K4me1/2^1,12–14^. SETD1A and SETD1B can catalyze H3K4me1/2/3. SET and MYND domain containing 1 (SMYD1/KMT3D) catalyze di- and trimethylation of H3K4, and another H3K4 methyltransferase, SET7/9, catalyzes H3K4me1^1,15,16^.

H3K4 methylation is removed via various demethylases. There are six main H3K4 demethylases currently known to be expressed in humans: two KDM1 family members (lysine-specific demethylase 1 (LSD1)/KDM1A and LSD2/KDM1B) and four KDM5 family members (jumonji AT-rich interactive domain 1a (JARID1A)/KDM5A, JARID1B/KDM5B, JARID1C/KDM5C, and JARID1D/KDM5D)^17^. Members of the KDM1 family specifically remove H3K4me1/2 and H3K9me1/2^18^, whereas KDM5 family enzymes are capable of removing H3K4me1/2/3^1^. Another JmjC domain-containing protein, ribosomal oxygenase 1 (RIOX1)/nucleolar protein 66 (NO66), was found to remove H3K4me1/2/3^19^.

### Phosphorylation-dependent stability of the Set1 family members MLL1 and MLL4

The relationship between H3K4 methylation dynamics and MLL1 stability was first discovered in the context of the cell cycle^20^. The degradation of MLL1 was found to be dependent on the Skp1-Cullin1-F-box-Skp2 (SCF^Skp2^) E3 ligase complex in the S phase of the cell cycle and on the APC/C^Cdc20^ complex in the late M phase (Fig. 4). Compensation for the degradation-mediated deficiency of the MLL1 protein by MLL1 overexpression was found to inhibit S phase progression. Conversely, disruption of DNA replication was shown to block MLL1 degradation, thereby activating the S phase checkpoint. In response to genotoxic (DNA-damaging) stress in the S phase, the DNA replication checkpoint kinase ataxia telangiectasia and rad3 related (ATR) phosphorylates serine 516 (S516) in MLL1^21^. The resulting S516-phosphorylated MLL1 is then stabilized, loses its physical interaction with SCF^Skp2^, and accumulates H3K4 methylation at the late replication origin, thereby inhibiting cell division cycle 45 (CDC45) loading and delaying DNA replication (Fig. 5a). These findings indicate that the control of MLL1 degradation via genomic integrity performs a crucial function in cell cycle progression. In contrast, MLL1 fusion proteins expressed in leukemic cells lack their C-terminal SET domain and are refractory to the SCF^Skp2^ –mediated degradation due to impaired interaction. Moreover, stable binding of MLL fusion proteins to chromatin prevents the accumulation of wild-type MLL1 and fails to promote H3K4 methylation. This implies that the escape of MLL1 fusion proteins from cell cycle- and genome integrity-dependent degradation elicits a pathogenic insult linked to leukemic development.

MLL1 proteins are subject to taspase1-dependent cleavage^22^. Genetic knockout (KO) of taspase1 in human cancer cells was shown to increase MLL1 stability, an effect that was partially dependent on proteasome activity^23^. The resulting stabilization of MLL1 in taspase1-KO cells resulted in an increase in its chromatin occupancy. Most, if not all, MLL1 fusion proteins lack the taspase1 cleavage site^23^ and are known to recruit the super elongation complex (SEC) to facilitate the transcription of oncogenes^24^. The expression of equal amounts of wild-type MLL1 and an MLL1–ALL1-fused gene from chromosome 4 protein (AF4) fusion protein, a pathogenic form found in leukemic cells, in taspase1-KO cells was found to increase the chromatin occupancy of wild-type MLL1 while reducing the binding of elongation factor for RNA polymerase II 2 (ELL2), an SEC subunit, at the same regions^23^. These findings suggest that uncleaved MLL1 is able to displace MLL1 fusion proteins from oncogenes. MLL1 contains a consensus casein kinase 2 (CK2) recognition sequence near the taspase1 cleavage site, and CK2 has been shown to phosphorylate threonine 2724 (T2724) and S2726 within this sequence. Like taspase1 loss, genetic ablation or pharmacological inhibition of CK2 was found to abrogate MLL1 cleavage and increase the MLL1 protein level. In addition, CK2 inhibitors were found to delay the progression of leukemia in MLL1-AF9 model mice and prolong their survival. These findings show that targeting MLL1 protein stability may be a promising therapeutic approach for treating leukemia associated with MLL translocations.

The stability of the MLL4 protein, another member of the Set1 family, was shown to be dependent on the F-box protein F-box and WD repeat domain containing 7 (FBXW7), which is a component of the E3 ligase complex SCF^Fbxw7^^25^. The interaction between FBXW7 and the N-terminal region of MLL4, which contains 25 SPPXE motifs, was found to be required for the proteasomal degradation of MLL4. Furthermore, compromised phosphorylation of MLL4 at its N-terminal region ablated MLL4 binding to FBXW7. These findings demonstrate that the phosphorylatable N-terminal region in MLL4 is involved in FBXW7-mediated degradation. The frequent occurrence of loss-of-function (LOF) mutations of MLL4 in diffuse large B-cell lymphoma (DLBCL) suggests that MLL4 functions as a tumor suppressor in DLBCL^26,27^. Conversely, FBXW7 expression was found to be elevated in samples from patients with multiple myeloma (MM) and DLBCL^25^. FBXW7 KO in DLBCL cells was shown to reprogram the transcription of genes in the oxidative phosphorylation pathway and impair proliferation. Concurrent loss of MLL4 reversed this transcriptional change and the proliferation defect induced by FBXW7 deletion. These observations imply that FBXW7-mediated MLL4 degradation promotes tumorigenic proliferation and that interfering with this axis may constitute a therapeutic approach for suppressing B-cell malignancies.

### Changing the cell state through the dynamic regulation of LSD1 stability

Proteasomal degradation of the zinc finger family protein LSD1 was first observed in cells lacking the REST corepressor 1 (CoREST) complex, which is essential for LSD1-nucleosome interactions^28^. The physical interaction of LSD1 with CoREST not only facilitates the binding of LSD1 to nucleosome substrates but also protects LSD1 from proteasomal degradation^28^. The E3 ligase responsible for LSD1 degradation when LSD1 is dissociated from CoREST remains unidentified. Han et al. reported that the plant homeodomain (PHD) finger protein jade family PHD finger 2 (JADE2) promotes LSD1 proteasomal degradation during neural differentiation^29^. Knockdown of JADE2 in embryonic stem cells (ESCs) was shown to inhibit LSD1 degradation. This effect was accompanied by decreased accumulation of H3K4 methylation on neurogenesis-related LSD1 target genes and reduced emergence of neural progenitors and mature neurons from JADE2-depleted ESCs in neural differentiation medium. These findings reveal that JADE2-mediated LSD1 degradation performs a critical function in promoting the differentiation of ESCs into neural cells. However, this study did not define the relationship between JADE2 and the LSD1–CoREST interaction.

Diverse DUBs contribute to LSD1 overexpression, a phenomenon frequently observed in various cancers^30–33^. The first DUB found to target LSD1 was the ubiquitin-specific peptidase ubiquitin specific peptidase 28 (USP28)^34^. Wu et al. reported a positive correlation between the USP28 and LSD1 protein levels in cancer cell lines and breast tumor samples. The expression of both USP28 and LSD1 was also found to be elevated in tumors from mouse mammary tumor virus-Wnt family member 1 (MMTV-Wnt1) transgenic mice compared with normal mammary glands. In support of these findings, USP28 knockdown impaired the self-renewal capacity of cancer stem cells (CSCs) derived from MMTV-Wnt1 tumors and promoted their differentiation. These findings highlight the critical role of USP28-mediated LSD1 stabilization in maintaining CSC-like properties and driving tumorigenicity.

In glioblastoma, the deubiquitinase USP22 facilitates LSD1 stabilization in a glycogen synthase kinase 3β (GSK3β)-dependent manner^35^. Phosphorylation of LSD1 at S683 by GSK3β was shown to promote the interaction of LSD1 with USP22, leading to LSD1 stabilization. Importantly, the protein levels of USP22, GSK3β, and LSD1 were found to be positively correlated with one another and concurrently increased in the nuclei of glioblastoma and glioma stem cells (GSCs). Depletion of USP22 or GSK3β destabilized LSD1, thereby suppressing the expression of stem cell markers and increasing the expression of differentiation markers. These findings demonstrate that nuclear GSK3β- and USP22-mediated LSD1 stabilization promotes GSC stemness and glioblastoma tumorigenesis.

More recently, DUB-mediated LSD1 stabilization has been suggested to contribute to breast cancer metastasis. Elevated protein levels of both the arginine methyltransferase coactivator associated arginine methyltransferase 1 (CARM1) and LSD1 were detected in human breast tumor samples, and increased levels of those proteins were correlated with increased tumor grade^36^. From a functional standpoint, the researchers further showed that the dimethylation of LSD1 at arginine 838 (R838) by CARM1 facilitates the binding of LSD1 to USP7, which, in turn, deubiquitinates and stabilizes LSD1. In breast cancer cells, treatment with a CARM1 inhibitor or expression of a CARM1-methylation-resistant LSD1 mutant inhibited cell migration and invasion. Conversely, overexpression of CARM1 or LSD1 in breast cancer cells promoted cell migration and invasion. Additionally, Gong et al. reported that ovarian tumor deubiquitinase 7B (OTUD7B) stabilizes LSD1, influencing breast cancer metastasis^37^. This group revealed elevated levels of both the OTUD7B and LSD1 proteins in breast cancer patients and reported that these elevated levels were associated with advanced tumor stage, lymph node metastasis, and poor overall survival outcomes. Mechanistically, they showed that OTUD7B removes K63-linked ubiquitin chains from lysine 226 and 277 (K226/K277) of LSD1 and demonstrated that the resulting OTUD7B-mediated deubiquitination stabilizes LSD1 and promotes the formation of the LSD1-CoREST complex, which drives the expression of genes involved in metastasis. Knockdown of either OTUD7B or LSD1 significantly reduced the expression of metastasis-associated genes and effectively suppressed the metastasis of breast cancer cells.

### Degradation of KDM5 family proteins in response to cellular stress

KDM5A stability has been linked to adult hippocampal neurogenesis (AHN), a process involving the generation of new neurons from neural stem cells during adulthood^38^. A significant reduction in AHN in the hippocampus has been reported in Alzheimer’s disease patients^39^. Kim et al. demonstrated that mitochondrial dysfunction induced by amyloid-beta expression triggers KDM5A degradation in neural progenitor cells^40^. The resulting loss of KDM5A was found to suppress the expression of genes associated with neurogenesis, thereby inhibiting the differentiation of neural progenitor cells. In a related study, KDM5A-knockdown mice presented a reduction in mature neurons in the subgranular zone and displayed impairment of cognitive function and spatial memory, highlighting the critical role of KDM5A stability in AHN.

KDM5A degradation has also been investigated in the context of breast cancer. F-box protein 22 (FBXO22) was identified as an E3 ligase responsible for KDM5A degradation in triple-negative breast cancer (TNBC)^41^. That study revealed that FBXO22 expression was lower in TNBC cells than in cells of other breast cancer subtypes and, conversely, that the KDM5A protein level was increased in TNBC cells. That study also revealed that ectopic expression of FBXO22 in TNBC cells inhibited their migration by decreasing the KDM5A protein level, which subsequently increased the expression of the FBXO22 target gene *cyclin-dependent kinase inhibitor 2A* (*CDKN2A)*, encoding p16. These findings highlight a role for FBXO22-mediated KDM5A degradation in preventing TNBC cell migration. On the other hand, tripartite motif containing 11 (TRIM11), identified by Xiao et al. as an E3 ligase responsible for KDM5C degradation^42^, was shown to be highly expressed in tissues from breast cancer patients, where its protein level was negatively correlated with the KDM5C protein level. This study further revealed that TRIM11 knockdown suppressed breast cancer cell proliferation and migration and that TRIM11 deficiency in animal models increased the KDM5C level and suppressed breast tumor growth. Collectively, these findings indicate that KDM5C degradation via TRIM11 promotes breast cancer cell proliferation and migration.

## Protein stability of H3K9 methylation modifiers

Mammalian cells express dozens of H3K9 methyltransferases containing catalytic SET domains flanked by pre-SET and post-SET domains; these include su(var)3-9 homolog 1 (SUV39H1)/KMT1A, SUV39H2/KMT1B, SET domain bifurcated histone lysine methyltransferase 1 (SETDB1)/KMT1E, G9a/euchromatic histone lysine methyltransferase 1 (EHMT1)/KMT1C, G9a-like protein (GLP)/EHMT2/KMT1D and several PRD-BF1 and RIZ homology domain containing (PRDM) family members^43^. H3K9 demethylation is carried out by the members of three families of mammalian H3K9 demethylases, namely, jumonji domain containing histone demethylase-2 (JHDM2)/KDM3, JHDM3(JMJD2)/KDM4 and PHF subfamily proteins, including KIAA1718/KDM7A, PHD finger protein 2 (PHF2)/KDM7C and PHD8/KDM7B, which harbor conserved catalytic JmjC domains.

### Oxygen-dependent regulation of the H3K9 modifiers G9a and SETDB1

**Fig. 3:**
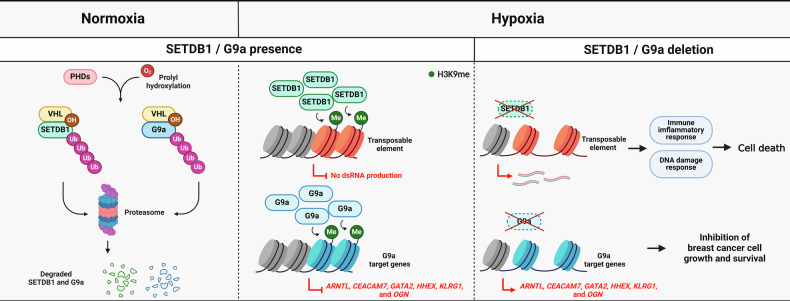
Oxygen-dependent regulation of SETDB1 and G9a. Schematic illustration of the oxygen-dependent functional regulation of SETDB1 and G9a. Left: In normoxia, PHDs hydroxylate proline residues of SETDB1 and G9a, enabling their VHL-mediated polyubiquitination and subsequent proteasomal degradation. Middle: In hypoxia, unhydroxylated SETDB1 and G9a are stabilized and protected from VHL-mediated degradation. The resulting increases in the SETDB1 and G9a levels lead to increased SETDB1 occupancy at chromatin associated with TEs and subsequent strong repression of TEs through H3K9 methylation. They also result in increased G9a occupancy at chromatin associated with its target tumor suppressor genes in breast cancer cells, leading to increased cell growth and survival. Right: Loss of SETDB1 in hypoxia results in failure of TE silencing, thereby activating TE transcript-driven immune-inflammatory and DNA damage responses, followed by cell death. G9a loss in hypoxia derepresses the transcription of G9a target genes, inhibiting the growth and survival of breast cancer cells.

Hypoxia, resulting from oxygen deficiency, is intimately associated with diverse physiological and pathological processes^44^. Reorganization of histone lysine methylation patterns on chromatin is among the robust responses to hypoxia. In addition to directly modulating the catalytic activity of histone lysine methylation modifiers, especially demethylases^45^, hypoxia regulates the stability of two different H3K9 methyltransferase proteins (Fig. 3).

Hypoxia-induced H3K9me2 accumulation associated with an increase in the protein level of the histone methyltransferase G9a was first reported more than a decade ago^46^. Thereafter, G9a was found to be hydroxylated at proline 676 and 1207 (P676/P1207) by the proline hydroxylases prolyl hydroxylase domain-containing protein 1 (PHD1) and PHD3^47^. Consistent with the observation that these proline hydroxylases utilize oxygen as a substrate, the cellular G9a hydroxylation level was found to decrease under hypoxia. Proteasomal degradation of hydroxylated G9a is mediated by its interaction with the tumor suppressor von Hippel-Lindau (VHL), a well-known E3 ligase adaptor for the CRL2^Vhl^ complex that recognizes and ubiquitinates hydroxylated proteins at proline residues^48^. Under hypoxic conditions, stabilized G9a escapes VHL-mediated degradation, increasing the H3K9me2 level at its target gene promoters and thereby downregulating the expression of its target genes. In breast cancer cells, G9a depletion was shown to relieve hypoxia-induced repression of a subset of tumor-suppressor genes. Furthermore, the suppressed expression of G9a target genes is significantly correlated with poor outcomes in breast cancer patients.

G9a forms a heterodimeric complex with another H3K9 methyltransferase, GLP. The E3 ligase speckle type BTB/POZ protein (SPOP) has been shown to physically interact with and ubiquitinate GLP, promoting its proteasomal degradation^49^. Interestingly, SPOP mutants harboring LOF mutations were found to stabilize GLP together with its partner protein, G9a, in prostate cancer (PCa) cells. The stabilized G9a/GLP complex increased DNA methylation by providing a binding platform for recruiting DNA methyltransferases to chromatin. Interestingly, this study demonstrated that the enzymatic activity of G9a/GLP is dispensable for increasing DNA methylation. Whether VHL-induced degradation of G9a is involved in mediating the stability of its partner GLP and the level of DNA methylation in PCa cells under hypoxic conditions warrants further investigation.

Given that SETDB1 has been implicated in several diseases, including cancers, neuropsychiatric disorders, congenital cardiovascular diseases and inflammatory bowel disease (IBD), SETDB1 has attracted considerable attention for its potential as a therapeutic target^50^. PHD-dependent proline hydroxylation of SETDB1 was recently reported in various cell types^51^. Under normoxic conditions, SETDB1 hydroxylated at P575, P755, and P1245 by PHDs was found to be recognized by the CRL2^Vhl^ complex and undergo ubiquitin-mediated degradation. In contrast, hypoxia stabilized SETDB1 by suppressing its hydroxylation. Transcripts derived from transposable elements (TEs) generate endogenous double-stranded RNAs that jeopardize genome stability^52^. Furthermore, this process, known as ‘viral mimicry’, enables endogenous retroviral (ERV) transcripts to activate antiviral signaling pathways related to the innate immune response^53^. Among H3K9 methyltransferases, SETDB1 is known to be critical for silencing TEs^54^. In a related observation, whereas G9a stabilization leads to transcriptional suppression of protein-encoding genes under hypoxic conditions^55^, SETDB1 stabilization is crucial for the transcriptional repression of TEs under hypoxic conditions. Moreover, the loss of SETDB1 activity under hypoxic conditions induces cell death associated with inflammation and DNA damage. The expression of innate immune-related genes and antiviral response genes is upregulated in patients with hypoxic tumors and low SETDB1 expression, suggesting that targeting SETDB1 to derepress TE-related innate immune responses may be a viable strategy for eliminating hypoxic cancer cells.

SETDB1 forms a heterodimer with activating transcription factor 7 interacting protein (ATF7IP)^56^. Two different studies reported that loss of ATF7IP reduces H3K9me3 modification at SETDB1 target loci, including TEs^57,58^. Timms et al. reported that, in addition to facilitating the nuclear localization of SETDB1, ATF7IP shields nuclear SETDB1 from polyubiquitination-mediated degradation^57^. This study revealed that the SETDB1 protein level is decreased mainly in the cytosol of ATF7IP-KO cells, although the E3 ligase responsible for SETDB1 degradation in the absence of ATF7IP was not identified.

### Cell cycle- and DNA damage-dependent stability of the H3K9 demethylases KDM4A and PHF8

**Fig. 4:**
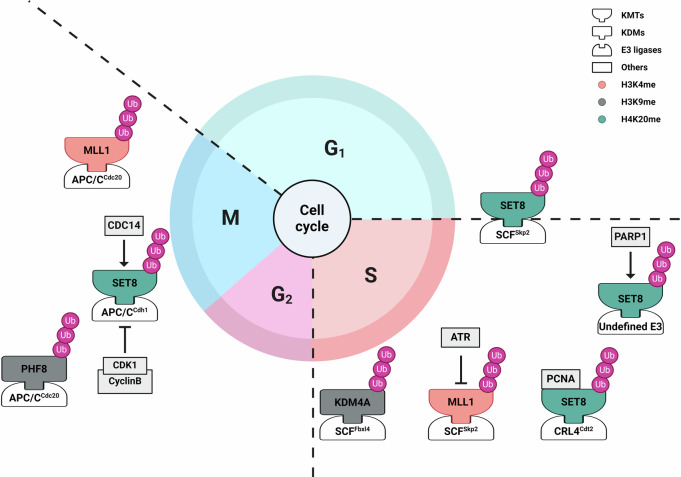
Regulation of KMT/KDM protein stability in a cell cycle-dependent manner. Schematic representation of histone lysine methylation modifiers regulated by cell cycle-dependent protein degradation. SET8 is degraded by SCF^Skp2^ at the G1/S transition and is dynamically regulated by CRL4^Cdt2^ during the S phase in a PCNA-dependent manner. PARP1-mediated poly(ADP-ribosyl)ation of SET8 promotes its UPS-dependent degradation. SET8 is also regulated in a phosphorylation-dependent manner during the G2/M phase by the CDK1/cyclin B complex, while the dephosphorylation of SET8 by CDC14 in the late M phase leads to its degradation by the APC/C^Cdh1^ complex. SCF^Fbxl4^-dependent proteasomal degradation accounts for the decrease in the KDM4A level in the S phase. PHF8 is regulated by the APC/C^Cdc20^ complex in the G2/M phase. MLL1 degradation is mediated by the E3 ligase complex SCF^Skp2^ in the S phase of the cell cycle and by the APC/C^Cdc20^ complex in the late M phase. In response to genotoxic stress during the S phase, the DNA replication checkpoint kinase ATR phosphorylates MLL1 and inhibits its degradation mediated by SCF^Skp2^. The different colors of the histone methylation modifiers indicate specific targets of the modifiers.

The UPS-dependent degradation of H3K9 demethylases is linked to cell cycle-related H3K9 methylation dynamics^59–61^. The four members (KDM4A–D) of the JHDM3/KDM4 family can remove H3K9me2/3 and H3K36me2/3^62–65^. During cell cycle progression, the KDM4A protein level is highest in the G1/S phase and then gradually decreases in the S phase, with the lowest level occurring during the G2/M phase^66^. Cullin1-dependent proteasomal degradation accounts for the decrease in the KDM4A level in the S phase (Fig. 4). Through a screen of small interfering RNAs (siRNAs) against F-box proteins, which are substrate recognition adaptors of the SCF E3 ligase complex, F-box and leucine-rich repeat protein 4 (FBXL4) was identified as the E3 ligase for S phase-dependent degradation of KDM4A. KDM4A overexpression was shown to accelerate S phase progression, accompanied by an increase in the number of replication forks. Conversely, forced degradation of KDM4A decreased the rate of DNA replication, as monitored by 5-bromo-2’-deoxyuridine (BrdU) incorporation. Although F-box proteins primarily recognize phosphorylated proteins as binding partners, whether the phosphorylation of KDM4A affects its SCF^Fbxl4^-dependent degradation is not yet known.

**Fig. 5:**
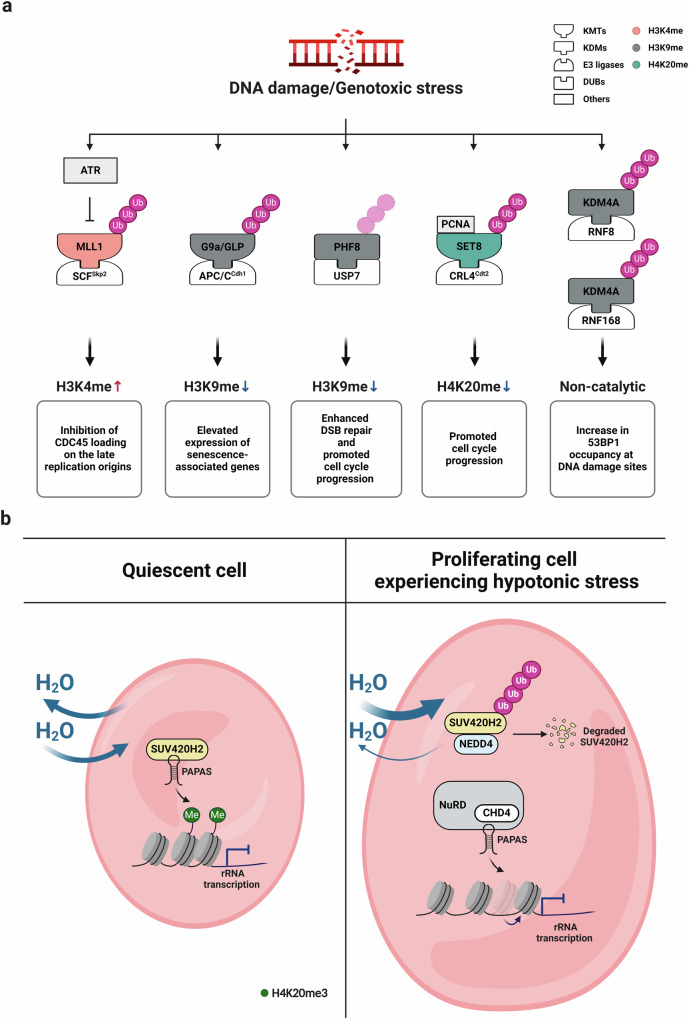
Regulation of KMT/KDM protein stability in response to cellular stress. Schematic representation of histone lysine methylation modifiers regulated in response to stress-dependent protein degradation. **a** DNA damage-induced degradation of histone methylation modifiers. In response to genotoxic stress, the DNA replication checkpoint kinase ATR phosphorylates MLL1. Phosphorylated MLL1 loses its physical interaction with SCF^Skp2^ and is thereby stabilized and accumulates H3K4 methylation at late replication origins, which inhibits CDC45 loading to delay DNA replication. DNA damage activates the APC/C^Cdh1^ E3 complex, enabling the binding of G9a as well as GLP. This binding triggers the proteasomal degradation of G9a and GLP, causing a global decrease in H3K9me2 and subsequently upregulating senescence-associated gene expression. Radiation-induced DNA damage promotes USP7-mediated stabilization of PHF8. The USP7-PHF8 axis promotes the cell cycle by decreasing the H3K9me1 levels on genes involved in the cell cycle. In addition, PHF8 accumulation at DNA damage sites promotes DSB repair. Upon DNA damage stress, the CRL4^Cdt2^ complex ubiquitinates and degrades SET8 in a PCNA-dependent manner. This leads to a decrease in H4K20 methylation to modulate the chromatin structure, promoting cell cycle progression. KDM4A is degraded by RNF8 and RNF168 in response to DNA damage, facilitating 53BP1 recruitment to DNA damage sites. The different colors of the histone methylation modifiers indicate specific targets of the modifiers. **b** Hypotonic stress-mediated stability of the SUV420H2 protein. The antisense RNA PAPAS mediates rDNA silencing via distinct mechanisms in cells exposed to different stresses. In quiescent cells, PAPAS is upregulated and guides the histone methyltransferase SUV420H2 to rDNA, leading to H4K20me3 and chromatin compaction. rRNA synthesis is subsequently attenuated. In contrast, in proliferating cells exposed to hypotonic stress, the SUV420H2 protein is degraded by the E3 ligase NEDD4. The resulting depletion of SUV420H2 facilitates the interaction of PAPAS with CHD4, a subunit of the NuRD complex, rather than with SUV420H2. Recruitment of the NuRD complex to rDNA through PAPAS modifies the promoter-bound nucleosome into a transcriptionally repressive state, thereby attenuating rRNA synthesis.

In response to DNA damage, KDM4A is degraded in a manner that depends on the RING finger proteins ring finger protein 8 (RNF8) and RNF168^67^ (Fig. 5a). Mallette et al. reported that the tandem Tudor domains within KDM4A compete with the p53-binding protein 1 (53BP1) for binding to H4K20me2 at DNA damage sites. In line with this observation, RNF8 and RNF168, which accumulate at DNA damage sites, resulted in the degradation of KDM4A, allowing 53BP1 to be recruited to support an efficient DNA damage response (DDR). These findings show that KDM4A stability is highly regulated by the UPS upon DNA damage.

PHF8, a homeodomain-containing zinc finger protein, is representative of another class of H3K9 demethylases. Cell cycle-dependent changes in the PHF8 protein level have been reported in HeLa cells, with the highest levels observed during the G2 phase and mitosis^68^. PHF8 contains an LXPKXL motif, which mediates its physical interaction with cell division cycle 20 (CDC20), a substrate recognition subunit of the E3 ligase complex APC/C^Cdc20^. The interaction between PHF8 and APC/C^Cdc20^ was shown to occur primarily during mitosis. APC/C^Cdc20^, which targets diverse cell cycle progression-related proteins for degradation, was demonstrated to mediate the polyubiquitination and subsequent proteasomal degradation of PHF8 (Fig. 4). This study further revealed that PHF8 occupancy of a subset of genes that are highly expressed in the G2 and M phases (G2/M), such as *cyclin B1* (*CCNB1)*, *non-SMC condensin I complex subunit G* (*NCAPG*) and *centromere protein A* (*CENPA*), was greater in the G2/M than in the G1 and S phases (G1/S). Consistent with these findings, less accumulation of H3K9me1/3 modifications in the region targeted by PHF8 was found in the G2/M than in the G1/S, whereas the expression of these genes in the G2/M was greater than that in the G1/S. In keeping with these findings, loss of PHF8 was shown to impair progression to the G2 phase and mitosis. Collectively, these findings suggest that PHF8, whose stability is regulated by the APC/C^Cdc20^ complex, plays a critical role in cell cycle functions important in the G2/M transition through the modulation of H3 methylation at specific chromatin regions.

The stability of the PHF8 protein also plays a crucial role in breast cancer^69^, with the cited study revealing that USP7-mediated deubiquitination stabilizes PHF8 and further demonstrating through transcriptome analysis and chromatin immunoprecipitation that the H3K9me1 level on genes involved in cell cycle regulation, including *cyclin A2* (*CCNA2*), is decreased and that the expression of these genes is increased via the USP7-PHF8 axis. Consistent with these findings, breast cancer cell proliferation was found to be promoted via the USP7-PHF8-Cyclin A2 axis. Radiation-induced DNA damage further promoted USP7-mediated stabilization of PHF8, increasing its level and allowing more PHF8 protein to be recruited to DNA double-strand break (DSB) sites (Fig. 5a). In contrast, efficient DNA repair failed in cells with depletion of either PHF8 or USP7. Bloom syndrome RecQ like helicase (BLM) and Ku70, both of which are essential for DSB repair, showed impaired recruitment at DSBs in cells deficient in PHF8 enzymatic activity, demonstrating that the lysine demethylase activity of PHF8 is crucial not only for the cell cycle but also for DSB repair.

### Stability of the H3K9 methylation modifiers G9a and GLP in senescent cells

The expression of genes encoding a series of secreted proteins, termed senescence-associated secretory phenotype (SASP) proteins, is increased in senescent cells. These proteins alter the local tissue environment and contribute to chronic inflammation. Takahashi et al. demonstrated that, in senescent cells, the DDR activates the APC/C^Cdh1^ E3 complex through both ATM-dependent and ATM-independent pathways^70^. They also showed that cadherin 1 (CDH1), the substrate recognition adaptor of APC/C^Cdh1^, enables the binding of G9a as well as GLP to the complex. This binding triggers the proteasomal degradation of G9a and GLP, thereby causing a global decrease in H3K9me2 associated with elevated expression of the *interleukin-6/8* (*IL-6/IL-8*) genes, encoding the major components of the SASP, in senescent cells (Fig. 5a). These findings delineate a pathway linking the DDR and histone lysine methylation to the modulation of gene expression in senescent cells.

### Degradation of the H3K9 methylation modifiers SUV39H1 and KDM3A in an acetylation-dependent manner

The nicotinamide adenine dinucleotide (NAD)-dependent deacetylase sirtuin 1 (SIRT1) has been shown to deacetylate SUV39H1 at K266, thereby increasing the H3K9me3-generating catalytic activity of SUV39H1^71^. Via a florescence recovery after photobleaching assay with the SUV39H1–enhanced green fluorescent protein (EGFP) fusion protein, Bosch-Presegué et al. found that SIRT1 promotes SUV39H1 turnover in constitutive heterochromatin^72^. Interestingly, they reported that this effect is not driven by the enzymatic activity of SIRT1. Instead, the physical interaction between SUV39H1 and SIRT1 increases the half-life of SUV39H1 by inhibiting the mouse double minute 2 homolog (MDM2)-dependent polyubiquitination of SUV39H1 at K87. Under stress conditions that lead to SIRT1 upregulation, such as oxidative stress and caloric restriction, the SUV39H1 level is increased in a SIRT1-dependent manner. These results demonstrate that the regulation of SUV39H1 protein stability is a key event influencing the modulation of heterochromatin structure in the context of cellular stress.

Xu et al. reported that KDM3A protein stability is controlled by the E3 ligase STIP1 homology and U-box containing protein 1 (STUB1) in castration-resistant prostate cancer (CRPC)^73^. They showed that the acetyltransferase p300 acetylates KDM3A at K421, enabling it to recruit the bromodomain and extraterminal domain (BET) family member bromodomain containing 4 (BRD4). The physical interaction between acetylated KDM3A and BRD4 was found to block STUB1-dependent KDM3A degradation and promote KDM3A recruitment to androgen receptor (AR) targets. Increased levels of both total and K421-acetylated KDM3A have been reported in prostate cancer cells resistant to the AR antagonist enzalutamide. Treatment of enzalutamide-resistant prostate cancer cells with inhibitors of either p300 or BET was shown to destabilize KDM3A, conferring sensitivity to the AR antagonist. These results demonstrate that inhibitors of p300 or BET may be efficacious in overcoming CRPC, at least in part by promoting STUB1-induced KDM3A degradation.

## Protein stability of H3K27 methylation modifiers

In mammalian cells, H3K27 methylation is catalyzed solely by polycomb repressive complex 2 (PRC2)^74^. PRC2 consists of the core subunits embryonic ectoderm development (EED), suppressor of zeste 12 homolog (SUZ12) and retinoblastoma binding proteins 4 and 7 (RBBP4/7); a catalytic subunit, either enhancer of zeste homolog 1 (EZH1)/KMT6B or EZH2/KMT6A; and a wide range of substoichiometric subunits^75^. Both EZH1 and EZH2 catalyze H3K27me1/2/3. Removal of H3K27 methylation is mediated by the KDM6 family members ubiquitously transcribed tetratricopeptide repeat protein X-linked (UTX)/KDM6A, jumonji domain-containing protein-3 (JMJD3)/KDM6B, and ubiquitously transcribed tetratricopeptide repeat containing Y-linked (UTY)/KDM6C^76^.

### Regulation of EZH2 degradation by diverse mechanisms

A number of studies have reported UPS-dependent EZH2 degradation by several E3 ligases, including SMAD-specific E3 ubiquitin protein ligase 2 (Smurf2), C-terminus of Hsc70-interacting protein (CHIP), ubiquitin-protein ligase E3 component N-recognin 4 (UBR4), praja ring finger ubiquitin ligase 1 (PRAJA1), beta-transducin repeat containing E3 ubiquitin-protein ligase (β-TrCP), TNF receptor-associated factor 6 (TRAF6), the proto-oncogene casitas B-lineage lymphoma (c-Cbl), and F-box and WD repeat domain containing 7 (FBW7). EZH2 deubiquitination is also regulated by several DUBs, including zinc finger RANBP2-type containing 1 (ZRANB1), USP7, USP21, USP36, USP44, and ubiquitin C-terminal hydrolase L1 (UCHL1). In addition, extensive studies have shown that diverse PTMs are involved in UPS-dependent EZH2 degradation. For details on findings on EZH2 protein stability accumulated in recent decades, we refer readers to a recent review by Guo et al.^77^. Here, we summarize the findings of recent reports that are not covered in the review.

UBR4 binding to the N-terminal domain of EZH2 promotes polyubiquitination-mediated EZH2 degradation in a variety of cell types^78,79^. UBR4-mediated EZH2 degradation has also recently been reported in melanoma cells^80^. It has been suggested that the melanin content, represented by high-pigmented cells (HPCs) and low-pigmented cells (LPCs), is a major source of the cellular heterogeneity in melanoma. The EZH2 protein level is elevated in LPCs, which exhibit more aggressive malignant behaviors. A decrease in the EZH2 protein abundance in pigmented melanoma cells alters the phenotypes of LPCs to those of HPCs and thereby suppresses tumorigenic proliferation. UBR4-induced ubiquitination of EZH2 at K381 is involved in the suppression of genes involved in melanin biosynthesis pathways, such as *oculocutaneous albinism type II* (*OCA2*), in LPCs.

Another recent study revealed that the E3 ligase thyroid hormone receptor interactor 12 (TRIP12) triggers K63-linked polyubiquitination of EZH2 at K634 and stabilizes EZH2 in extranodal natural killer/T-cell lymphoma (ENKTL)^81^. Furthermore, ubiquitination of EZH2 promotes the interaction between EZH2 and SUZ12 and increases cyclin-dependent kinase 1 (CDK1)-induced phosphorylation of EZH2 at T487, resulting in EZH2 stabilization. Moreover, the TRIP12-EZH2 axis was found to mediate ENKTL cell migration via the induction of epithelial-mesenchymal transition (EMT). These findings reveal a mechanistic link between EZH2 and TRIP12 overexpression in ENKTL, suggesting that the TRIP12-EZH2 axis might be a potential therapeutic target for suppressing ENKTL metastasis.

### Regulation of UTX stability in cancer progression

The histone demethylase UTX is generally regarded as a tumor suppressor, consistent with the recurrent finding of UTX LOF mutations in various cancers^82^. MLL3/4 complexes associate with UTX to activate enhancers^83^. UTX-mediated H3K27 demethylation is crucial for generating unmodified H3K27 residues for acetylation (H3K27ac), an active enhancer mark^84^. Concurrent H3K4 methylation and H3K27 demethylation by MLL3/4 complexes cooperate in the regulation of gene expression by establishing active enhancers. Interestingly, MLL4 loss in multipotent mouse cells was found to result in destabilization of the UTX protein^83^. This finding raises the possibility that the incorporation of UTX into the MLL4 complex prevents its degradation, although the mechanism was not investigated in detail in that study.

In addition to LOF of UTX, low UTX expression is associated with cancers^85^. The transcriptional downregulation of UTX is often linked to cancer progression^86^. Recently, UTX protein degradation was shown to be dysregulated in colorectal cancer (CRC), underscoring the importance of UTX protein stability^87^. The CRL4B^Cop1^ E3 complex induces UPS-dependent UTX protein degradation in CRC cells. Moreover, in mouse models, the deficiency of constitutive photomorphogenic protein 1 (COP1) promotes CRC, in part through UTX stabilization. In support of these findings, immunohistochemical analysis of CRC tissue samples revealed an inverse correlation between the COP1 and UTX protein levels.

## Protein stability of H3K36 methylation modifiers

In mammals, the methyltransferases responsible for H3K36 methylation include nuclear receptor binding SET domain protein 1 (NSD1)/KMT3B, NSD2/KMT3G, NSD3/KMT3F, ASH1-like histone lysine methyltransferase (ASH1L)/KMT2H, SMYD2/KMT3C, SET domain-containing protein 2 (SETD2)/KMT3A, SETD3 and SET domain and mariner transposase fusion gene (SETMAR). Among these methyltransferases, NSD3 catalyzes only H3K36me1; NSD1, NSD2, ASH1L, SMYD2, SETD3 and SETMAR catalyze H3K36me2; and SETD2 is specialized for catalyzing H3K36me3^88,89^.

There are two H3K36 demethylase families in mammals: JHDM1/KDM2A-B and JHDM3/KDM4A-D. KDM2A-B family members can catalyze H3K36me1 and H3K36me2, and KDM4 family members can remove H3K36me2 and H3K36me3^64,90–92^.

### Phosphorylation-dependent regulation of NSD2 stability

NSD2 is considered an oncogene, consistent with its overexpression and mutation in many cancers^93,94^. Recent studies have revealed that phosphorylation plays a critical role in modulating cellular NSD2 stability. For example, in phosphatase and tensin homolog (PTEN)-null prostate cancer cells, Akt serine/threonine kinase (AKT) interacts with and directly phosphorylates NSD2 at S172, preventing NSD2 degradation by the CRL4–chromatin licensing and DNA replication factor 1 (CRL4^Cdt1^) complex^95^. Prostate cancer cells expressing an NSD2 mutant in which S172 is replaced with alanine (S172A) exhibit significant impairment of both migration and anchorage-independent growth. In addition, mouse xenograft models established with prostate cancer cells carrying the NSD2 S172A mutant alleles exhibit substantially reduced numbers of metastatic lesions in the limbs, underscoring the crucial role of AKT-mediated phosphorylation and subsequent stabilization of NSD2 in prostate cancer metastasis.

In contrast to phosphorylation, dephosphorylation was demonstrated to promote the destabilization of NSD2. During dendritic cell (DC) activation, protein phosphatase 2Cδ (PP2Cδ), a serine/threonine phosphatase, binds to and dephosphorylates NSD2^96^. The phosphatase activity of PP2Cδ is critical for facilitating the interaction between NSD2 and the CRL4–DDB1 and CUL4 associated factor 2 (CRL4^Dcaf2^) complex, which promotes the ubiquitination and subsequent proteasomal degradation of NSD2. NSD2 degradation mediated by PP2Cδ and the CRL4^Dcaf2^ complex is crucial for decreasing the H3K36me2 level in the promoter of *RPTOR-independent companion of MTOR complex 2* (*RICTOR*). This degradation pathway suppresses mTORC2 signaling and DC activation, contributing to immune homeostasis.

In MM cells, aurora kinase A (Aurora A) directly interacts with and phosphorylates NSD2 at S56, thereby protecting NSD2 from cleavage and degradation^97^. NSD2 stabilized by Aurora A contributes to chemoresistance by increasing the expression of anticancer drug resistance-related genes via H3K36me2. Although the specific E3 ligase responsive to phosphorylation of NSD2 in MM remains unidentified, breast cancer 1 (BRCA1) was found to act as an E3 ligase to support NSD2 degradation in the human myelogenous leukemia cell line K562^98^. The involvement of NSD2 in suppressing cellular differentiation has been demonstrated in the hematopoietic and neuronal lineages^99,100^. During hemin-mediated differentiation of K562 cells, BRCA1 triggers the degradation of NSD2 through its ubiquitination at K292. Mutations in BRCA1, including K1183R, are frequently detected in cancers^101^. Interestingly, in hemin-treated K562 cells, BRCA1 K1183R failed to localize to the nucleus and decrease the NSD2 protein level. Moreover, cells expressing the BRCA1 K1183R mutant presented differentiation defects. These observations suggest that the BRCA1‒NSD2 axis is important in regulating the differentiation of leukemic cells.

### Regulation of transcription-coupled mRNA splicing through control of SETD2 protein stability

SETD2-mediated H3K36me3 modification is associated with transcription-coupled mRNA splicing and DNA damage responses^102,103^. In mammalian cells, a low level of the SETD2 protein is maintained through proteasomal degradation^104,105^. The overexpression of SPOP, identified as an E3 ligase for SETD2^104^, was shown to reduce the H3K36me3 level in genes that are subject to alternative mRNA splicing, such as *pyruvate kinase M 2 (PKM2)*, *tropomyosin 1 (TPM1)* and *TPM2*; conversely, the deletion of SPOP increased the H3K36me3 level in these genes. These findings suggest that SPOP-mediated SETD2 degradation acts as a regulatory switch to control gene expression through alternative splicing. The N-terminal region (amino acids 1–1403) of SETD2 facilitates its proteasome-mediated degradation^106^. The mRNA expression of full-length SETD2 (SETD2 FL) and an N-terminal deletion mutant of SETD2 (SETD2 C) at the same level was shown to result in an increased level of the SETD2 C protein compared with the SETD2 FL protein. This increase in the SETD2 C protein level increased H3K36me3, leading to increased expression of cell cycle-related genes and altered mRNA splicing of genes involved in both the cell cycle and RNA splicing. SETD2 C expression also promoted cell proliferation, migration, and invasion. Collectively, these findings highlight the importance of N-terminal domain-mediated degradation of SETD2 in maintaining its proper function and ensuring its ability to regulate the fidelity of transcription and splicing-related processes.

## Protein stability of H3K79 methylation modifiers

Disruptor of telomeric silencing-1 like histone lysine methyltransferase (DOT1L)/KMT4 is responsible for H3K79me1/2/3 in actively transcribed genes^107^. In particular, DOT1L has a well-documented role in MLL-fusion leukemia with aberrant rearrangement of MLL1 alleles. MLL fusion partner proteins, such as AF10, AF9, and eleven nineteen leukemia (ENL), interact directly with DOT1L, which plays a role in the overactivation of MLL target genes through H3K79 methylation^108^.

### Regulation of DOT1L stability in cancer

Control of DOT1L stability is a crucial regulatory mechanism that impacts various cellular processes, including gene expression and malignant progression. DOT1L stability has been shown to be regulated through various PTMs. For example, Liu et al. demonstrated that DOT1L stability is regulated by CREB-binding protein (CBP)-mediated acetylation^109^. That study revealed that DOT1L is acetylated at K358 in CRC cells, a modification that is positively correlated with CRC stage. Acetylation of DOT1L at K358 inhibits the binding of the E3 ligase RNF8, thereby preventing its proteasomal degradation. This stabilization enables DOT1L to catalyze H3K79 methylation of genes involved in EMT, promoting cancer metastasis and invasion. Consistent with this, the expression of an acetylation mimetic mutant of DOT1L was found to induce a cancer-like phenotype in vitro, further emphasizing the role of DOT1L acetylation in CRC progression.

In another study, Song et al. reported that DOT1L was stabilized in response to external environmental signals^110^, showing that the extracellular glucose level impacts DOT1L stability through the hexosamine biosynthetic pathway (HBP). Specifically, DOT1L was found to undergo O-GlcNAcylation at the C-terminal residue S1511 via the HBP. This O-GlcNAcylation was shown to promote the stability of DOT1L by inhibiting its interaction with the E3 ligase ubiquitin-protein ligase E3 C (UBE3C). DOT1L stabilized by glucose metabolism was found to regulate the expression of *homeobox A9 (HOXA9)* and *meis homeobox 1* (*MEIS)*, an effect associated with the proliferation of MLL-fusion leukemia. Inhibiting key players in the HBP or blocking O-GlcNAc transferase activity was demonstrated to increase cellular sensitivity to DOT1L inhibition, suggesting that targeting DOT1L degradation is a potential therapeutic strategy for MLL-fusion leukemia.

## Protein stability of H4K20 methylation modifiers

In mammals, different types of H4K20 methylation are catalyzed by specific methyltransferases. Among these, SET8/PR-SET7/KMT5A catalyzes H4K20me1, whereas SUV420H1/KMT5B and SUV420H2/KMT5C catalyze H4K20me2 and H4K20me3, respectively^111^. H4K20me1 and H4K20me2 influence DNA replication and DNA damage repair, whereas H4K20me3 is a marker of silenced heterochromatin^112^.

Several distinct demethylases are involved in the removal of H4K20 methylation. While PHF2 and PHF8 function as H3K9 demethylases, they can also function as H4K20 demethylases^113,114^. Another study revealed that LSD1n, an alternatively spliced form of LSD1 expressed exclusively in neurons, exhibits demethylase activity toward H4K20me1 and H4K20me2 in vitro^115^.

### Dynamics of SET8 degradation during the cell cycle

The mechanisms governing SET8 stability and its pivotal role in controlling cell cycle transitions have been explored in several studies (Fig. 4). Yin et al. provided the first such report in 2008, demonstrating that SET8 is degraded by SCF^Skp2^ at the G1/S transition^116^. SET8 protein stability is also dynamically regulated by the CRL4–cdc10-dependent transcript 2 (CRL4^Cdt2^) complex^117,118^ and the APC/C^Cdh1^ ^119^ complex during the cell cycle. During S phase or upon DNA damage stress, the CRL4^Cdt2^ complex ubiquitinates and degrades SET8 in a proliferating cell nuclear antigen (PCNA)-dependent manner. Inactivation of CRL4^Cdt2^ leads to SET8 stabilization, which results in aberrant H4K20me1 accumulation and chromatin compaction, triggering cell cycle arrest in the G2 phase (Fig. 5a)^117,118^. On the other hand, SET8 stability in the G2/M is regulated in a phosphorylation-dependent manner^119^, specifically by CDK1/cyclin B complex-mediated phosphorylation at S29 of SET8. Phosphorylated SET8 fails to interact with the APC/C^Cdh1^ complex and thus is protected from proteasomal degradation. Conversely, dephosphorylation of SET8 by CDC14 in the late M phase leads to SET8 degradation by APC/C^Cdh1^, which contributes to cell cycle progression. SET8 also interacts with poly(ADP-ribose) polymerase 1 (PARP1) in a cell cycle-dependent manner^120^. The consequent PARP1-mediated poly(ADP-ribosyl)ation of SET8 promotes its UPS-dependent degradation, highlighting another regulatory layer in the control of SET8 stability.

### SUV420H2 degradation in response to hypotonic stress

Ribosomal RNA (rRNA) synthesis is regulated to allow adaptations to specific environmental challenges^121,122^. In quiescent cells, the expression of the long noncoding RNA promoter and pre-rRNA antisense (PAPAS) is upregulated. PAPAS, in turn, facilitates the recruitment of SUV420H2 to ribosomal DNA (rDNA), resulting in transcriptional repression and chromatin compaction of the gene through H4K20me3 accumulation^123,124^. In proliferating cells, hypotonic stress increases the expression of PAPAS; however, in this case, the H4K20me3 level is not increased, but the abundance of pre-rRNA transcripts in these cells is nonetheless reduced^125^. The discrepancy between H4K20me3 occupancy and reduced pre-rRNA transcription is attributable to SUV420H2 protein degradation. Specifically, upon hypotonic stress, the SUV420H2 protein is degraded by the E3 ligase neuronal precursor cell-expressed developmentally downregulated 4 (NEDD4) (Fig. 5b). The resulting depletion of SUV420H2 facilitates the interaction of PAPAS with CHD4, a subunit of the nucleosome remodeling and deacetylase (NuRD) complex^125^. Recruitment of the NuRD complex to rDNA through PAPAS shifts the promoter-bound nucleosome into a transcriptionally repressive state. Under hypotonic conditions, H4K20me3-dependent chromatin compaction is insufficient to support robust suppression of rDNA transcription. In this context, NEDD4-dependent SUV420H2 degradation becomes necessary for PAPAS to select the NuRD complex as a binding partner and effectively terminate rDNA transcription. This finding implies that the regulation of SUV420H2 protein stability is involved in cellular homeostasis in response to stress.

## Therapeutic opportunities for targeting histone lysine methylation modifiers through protein degradation

The preceding sections provide a comprehensive summary of histone methylation modifiers and the mechanisms that regulate their stability. Here, we consider recently emerging novel approaches to drug development utilizing the UPS. An innovative drug development strategy, known as targeted protein degradation (TPD), induces the degradation of target proteins by promoting physical proximity between the target protein and an irrelevant E3 ubiquitin ligase^126^. This technology has been successfully employed to develop several degraders that target histone methylation modifiers. Innovative PRC2 degraders targeting the catalytic subunit EZH2 or the H3K27me3 binding partner EED have been reported and demonstrated promising antiproliferative effects in EZH2 gain-of-function mutant and TNBC cells by reducing the level of H3K27 methylation^127^. Additionally, it has been reported that heterobifunctional degraders targeting two H3K36 methyltransferases, NSD2 and NSD3, via binding proteins of the NSD N-terminal PWWP (PWWP1) domain result in a decrease in H3K36 methylation^127^. These degraders are still in the early developmental stage, and further optimization is necessary for their medical applications.

Interestingly, recent findings have suggested a potential therapeutic opportunity for controlling the stability of targets by harnessing the intrinsic proximity between the substrate and its endogenous E3 ligase. Ciulli et al. demonstrated that two degraders of the acetyl-lysine reader BRD4 forcibly stabilize the interactions between BRD4 and the E3 ligases DCAF11 and DCAF16, driving the efficient degradation of BRD4^128^. Both DCAF11 and DCAF16 are primed for BET bromodomain recognition and intrinsically possess a low affinity for BRD4, even in the absence of the compound. This finding suggests a novel strategy for developing a modality that stabilizes the interaction between the target protein and its cognate E3 ligase. For example, VHL controls the stability of two H3K9 methyltransferases, SETDB1 and G9a, in a proline hydroxylation-dependent manner. Thus, the ‘molecular glue’ that increases the affinity of VHL for SETDB1 or G9a may facilitate the degradation of SETDB1 or G9a regardless of oxygen availability.

## Concluding remarks

In this review, we highlighted the importance of the UPS-dependent regulation of histone lysine methylation modifiers in cell physiology and pathology (Table 1). The selectivity of E3 ligases and/or DUBs toward particular modifiers is the underlying mechanism that governs the degradation of modifiers. Moreover, diverse PTMs of modifiers differentially contribute to degradation processes. The observation that the PTMs of modifiers are associated with distinct signaling cascades suggests that targeting these signaling cascades provides an opportunity to modulate the stability of the related modifiers and thereby alter the epigenetic landscape. We anticipate that future studies employing various proteomic and genomic techniques, which are actively being improved, will reveal novel interactions between E3s/DUBs and histone lysine methylation modifiers and identify additional PTMs of these modifiers.

**Table 1:** Histone lysine methylation modifiers controlled by protein stability.

Histone lysine residue	Histone lysine modifiers	E3 ligases/DUBs	Regulators of protein degradation	Stimuli regulating protein degradation	Regulator’s roles in degradation	Biological function of protein stability	Disease connection	Ref.
H3K4	MLL1	SCF^Skp2^	ATR	Genotoxic stress in S phase	Genotoxic stress triggers ATR- dependent phosphorylation of MLL1 at S516, which block SCF^Skp2^-mediated MLL1 degradation	MLL1 stabilization delays DNA replication under DNA damaging stress	MLL-fusion leukemia	^20,21^
	MLL1	APC/C^Cdc20^		Late M phase	APC/C^Cdc20^ degrades MLL1 in late M phase	MLL1 degradation is necessary for proper cell cycle progression		^20^
	MLL1	undescribed	Taspase1, CK2		CK2 phosphorylates MLL1 at T2724/S2726, promoting taspase1- mediated MLL1 cleavage	MLL1 cleavage has a role in MLL1 activation and rapid turnover	MLL-fusion leukemia	^22,23^
	MLL4	FBXW7	N-terminal phospho-degrons of MLL4		Multiple N-terminal phosphodegrons of MLL4 are required to promote interaction with FBXW7	FBXW7-mediated MLL4 degradation promotes tumorigenic proliferation	DLBCL	^25^
	LSD1	undescribed	CoREST		Physical interaction of LSD1 with CoREST protects LSD1 from proteasomal degradation	LSD1-CoREST interaction not only facilitates LSD1 binding to nucleosome substrates but also protects LSD1 from proteasomal degradation		^28^
	LSD1	JADE2		Neurogenesis	JADE2 promotes LSD1 proteasomal degradation during neural differentiation	JADE2-mediated LSD1 degradation de-represses genes involved in neurogenesis and promotes differentiation of ESCs into the neural lineage	Neuroblastoma	^29^
	LSD1	USP28			USP28 stabilizes LSD1 via deubiquitination	LSD1 stabilization by USP28 suppresses gene expression involved in cellular differentiation and confers breast cancer cells with cancer stem-cell-like features	Breast cancer	^34^
	LSD1	USP22	GSK3β		Phosphorylation of LSD1 at S683 by GSK3β promotes the interaction of LSD1 with USP22, leading to LSD1 stabilization	Nuclear GSK3β- and USP22- mediated LSD1 stabilization is required for stemness of glioma stem cells and glioblastoma tumorigenesis	Glioma	^35^
	LSD1	USP7	CARM1		CARM1 di-methylates LSD1 at R838, facilitating LSD1 binding to USP7, which in turn deubiquitinates and stabilizes LSD1	CARM1-USP7 mediated LSD1 stabilization promotes migration and invasion of breast cancer cells	Breast cancer	^36^
	LSD1	OTUD7B			OTUD7B removes K63-linked ubiquitin chains from K226/K277 and stabilizes LSD1	OTUD7B-mediated deubiquitination stabilizes LSD1 and promotes the formation of the LSD1-CoREST complex, which drives the expression of genes involved in metastasis	Breast cancer	^37^
	KDM5A	undescribed		Mitochondrial dysfunction	Mitochondrial dysfunction triggers KDM5A degradation in neural progenitor cells	Degradation of KDM5A suppresses the expression of genes associated with neurogenesis, thereby inhibiting the differentiation of neural progenitor cells	Adult hippocampal neurogenesis	^40^
	KDM5A	FBXO22			FBXO22 degrades KDM5A	FBXO22-mediated KDM5A degradation induces *CDKN2A* gene expression, which inhibited metastasis in TNBC	TNBC	^41^
	KDM5C	TRIM11			TRIM11 degrades KDM5C	KDM5C degradation by TRIM11 promotes breast cancer proliferation and migration	Breast cancer	^42^
H3K9	G9a	CRL2^Vhl^	PHD1, PHD3	Oxygen	In normoxic condition, hydroxylation on P676 and P1207 residues of G9a promotes degradation by CRL2^Vhl^	In hypoxia, stabilized G9a drives hypoxia-mediated gene repression for breast cancer cell survival and tumorigenesis	Breast cancer	^47^
H3K9	G9a, GLP	SPOP			SPOP physically interacts with and ubiquitinates GLP for its proteasomal degradation	SPOP harboring LOF mutation stabilizes GLP along with its partner protein G9a in prostate cancer cells. The stabilized G9a/GLP complex upregulates DNA methylation by providing a binding platform for the recruitment of DNA methyltransferases to chromatin	Prostate cancer	^49^
	SETDB1	CRL2^Vhl^	PHDs	Oxygen	In normoxic condition, hydroxylation on P575, P755, P1245 residues of SETDB1 triggers degradation by CRL2^Vhl^	In hypoxia, SETDB1 stabilization is essential for TE repression to prevent hyperactivation of the immune inflammatory response and DNA damage-induced cell death		^51^
	SETDB1	undescribed	ATF7IP		ATF7IP shields nuclear SETDB1 from proteasomal degradation	ATF7IP plays an essential role in heterochromatin formation by shielding SETDB1 from proteasomal degradation in the nucleus	Colon cancer	^57^
	KDM4A	SCF^Fbxl4^		S phase	SCF^Fbxl4^ degrades KDM4A in S phase	KDM4A degradation is necessary for proper cell cycle progression		^66^
	KDM4A	RNF8, RNF168		DNA damage	Upon DNA damage, RNF8 and RNF168 degrade KDM4A	RNF8 and RNF168, which accumulate at DNA damage sites, eliminate KDM4A to allow the recruitment of 53BP1 for an efficient DNA damage response		^67^
	PHF8	APC/C^Cdc20^		Cell cycle	APC/C^Ccd20^ degrades PHF8	The downregulation of PHF8 leads to prolonged G2 phase and defective mitosis, resulting from the transcriptional inactivation of key G2/M genes during G2 phase		^68^
	PHF8	USP7			USP7 stabilizes PHF8	PHF8 stabilization is required for cell cycle progression and DSB repair	Breast cancer	^69^
	G9a, GLP	APC/C^Cdh1^		DNA damage	DDR induces proteasomal degradation of G9a and GLP through APC/C^Cdh1^	Degradation of G9a and GLP causes a global decrease in H3K9me2 associated with elevated expression IL-6/IL-8 genes, the major component of SASP in senescent cells		^70^
	SUV39H1	MDM2	SIRT1	Oxidative/metabolic stress	Physical interaction between SUV39H1 and SIRT1 increases SUV39H1 half-life by inhibiting its polyubiquitination at K87 by MDM2	K87 mutation increases SUV39H1 turnover in pericentromeric heterochromatin and protects genome integrity		^72^
	KDM3A	STUB1	p300		p300 acetylates KDM3A at K421, recruiting BRD4 to block STUB1- dependent KDM3A degradation	Interaction between acetylated KDM3A and BRD4 promotes KDM3A recruitment to AR targets, developing resistance to the AR antagonist enzalutamide	Castration- resistant prostate cancer	^73^
H3K27	EZH2	UBR4			UBR4 degrades EZH2	Downregulation of EZH2 in pigmented melanoma cells alters phenotypes of LPCs to those of HPCs and thereby suppresses tumorigenic proliferation	Melanoma cell	^78–80^
	EZH2	TRIP12	HSP60		TRIP12 triggers K63-linked polyubiquitination of EZH2 at K634 and stabilizes EZH2	TRIP12-mediated EZH2 stabilization accelerates ENKTL cell migration via inducing EMT	ENKTL	^81^
	UTX	undescribed	MLL4 loss		MLL4 loss in multipotent mouse cells induces destabilization of UTX protein	MLL3/4 complexes associate with UTX to activate enhancers		^83^
	UTX	CRL4B^Cop1^			CRL4B^Cop1^ degrades UTX	UTX degradation promotes CRC tumorigenesis	CRC	^87^
H3K36	NSD2	CRL4^Cdt1^	AKT		AKT phosphorylates NSD2 at S172, preventing CRL4^Cdt1^-mediated NSD2 degradation	Stabilization of NSD2 promotes *RICTOR, Rac1* gene expression to increase AKT signaling activation and prostate cancer cell motility	Prostate cancer	^95^
	NSD2	CRL4^Dcaf2^	PP2Cδ	Dendritic cell activation	PP2Cδ-mediated dephosphorylation of NSD2 promotes its interaction with the CRL4^Dcaf2^, leading to NSD2 degradation	NSD2 degradation suppresses mTORC2 signaling and DC activation	Immune system homeostasis	^96^
	NSD2	undescribed	Aurora A		Aurora A phosphorylates NSD2 at S56, protecting NSD2 from cleavage and degradation	NSD2 stabilized by Aurora A contributes to chemoresistance	Multiple myeloma	^97^
	NSD2	BRCA1			BRCA1 degrades NSD2 through ubiquitination of NSD2 at K292	BRCA1-mediated NSD2 degradation is required for erythroid differentiation	Differentiation of leukemic cancer	^98^
	SETD2	SPOP			SPOP degrades SETD2	SPOP-mediated SETD2 degradation acts as a regulatory switch to control gene expression through alternative splicing		^104^
	SETD2	undescribed	N-terminal region of SETD2		N-terminal region of SETD2 facilitates proteasome-mediated SETD2 degradation	N-terminal region-mediated SETD2 degradation is required for proper SETD2 function, regulating the fidelity of transcription and splicing- related processes		^106^
H3K79	DOT1L	RNF8	CBP		CBP acetylates DOT1L at K358, preventing RNF8-mediated DOT1L degradation	DOT1L stabilization catalyzes H3K79 methylation on genes involved in EMT, promoting cancer metastasis and invasion	CRC	^109^
	DOT1L	UBE3C	O-GlcNAc transferase	Extracellular glucose level and HBP	O-GlcNAcylation of DOT1L at S1511 promotes DOT1L stabilization by blocking its interaction with UBE3C	DOT1L stabilization activates *HOXA9* and *MEIS* gene expression and MLL-fusion leukemia proliferation	MLL-fusion leukemia	^110^
H4K20	SET8	SCF^Skp2^		G1/S transition	SET8 is degraded by SCF^Skp2^ at the G1/S transition	SET8 degradation is required for proper cell cycle transition		^116^
	SET8	CRL4^Cdt2^		S phase or DNA damage	During S phase or upon DNA damage stress, the CRL4^Cdt2^ degrades SET8 in PCNA-dependent manner	Inactivation of CRL4^Cdt2^ leads to SET8 stabilization, which results in aberrant H4K20me1 accumulation and chromatin compaction, triggering cell cycle arrest in G2		^117,118^
	SET8	APC/C^Cdh1^	CDK1/cyclin B	G2/M phase	CDK1/cyclin B complex phosphorylates SET8 at S29, which block APC/C^Cdh1^-mediated SET8 degradation	SET8 S29 phosphorylation results in removal of SET8 from mitotic chromosomes		^119^
	SET8	APC/C^Cdh1^	CDC14	Late M phase	CDC14-mediated dephosphorylation of SET8 at S29 in late M phase leads to its degradation by APC/C^Cdh1^	SET8 degradation is important for proper mitotic progression		^119^
	SET8	undescribed	PARP1	S phase	PARylation of SET8 not only compromises its catalytic activity but also promotes its degradation via ubiquitination pathway	PARP1 regulates H4K20me1/3 distribution in the genome		^120^
	SUV420H2	NEDD4		Hypotonic stress	Upon hypotonic stress, NEDD4 degrades SUV420H2 protein	The degradation of SUV420H2 under hypotonic stress conditions plays a crucial role in redirecting the function of PAPAS from promoting chromatin compaction to facilitating nucleosome repositioning		^125^

The functional links between the dysregulated stability of histone lysine methylation modifiers and various human diseases emphasize the importance of targeting the stability of these modifiers for clinical purposes. Interestingly, a subset of histone lysine methylation modifiers, including EZH2, SETD1A and LSD1, exhibits both catalytic and noncatalytic activities that are important in cell physiology^129–131^. Targeting these modifiers with catalytic inhibitors would leave their noncatalytic activities unaffected. In contrast, elimination of these modifiers by promoting their degradation would inhibit their catalytic and noncatalytic activities. Such considerations underscore the importance of a concerted research effort to determine the molecular mechanisms that regulate protein stability for the development of future therapeutic strategies. Accumulating knowledge about the dynamics of modifier protein stability will not only increase our understanding of cell physiology associated with the epigenetic landscape but also aid in the identification of novel drug targets for modulating histone lysine methylation events related to pathological processes.
